# Underwater sound from vessel traffic reduces the effective communication range in Atlantic cod and haddock

**DOI:** 10.1038/s41598-017-14743-9

**Published:** 2017-11-07

**Authors:** Jenni A. Stanley, Sofie M. Van Parijs, Leila T. Hatch

**Affiliations:** 1 National Oceanic and Atmospheric Administration, Northeast Fisheries Science Center, National Marine Fisheries Science Center, Protected Species Branch, Woods Hole, MA USA; 2 National Oceanic and Atmospheric Administration, National Ocean Service, Office of National Marine Sanctuaries, Stellwagen Bank National Marine Sanctuary, Scituate, MA USA

## Abstract

Stellwagen Bank National Marine Sanctuary is located in Massachusetts Bay off the densely populated northeast coast of the United States; subsequently, the marine inhabitants of the area are exposed to elevated levels of anthropogenic underwater sound, particularly due to commercial shipping. The current study investigated the alteration of estimated effective communication spaces at three spawning locations for populations of the commercially and ecologically important fishes, Atlantic cod (*Gadus morhua*) and haddock (*Melanogrammus aeglefinus*). Both the ambient sound pressure levels and the estimated effective vocalization radii, estimated through spherical spreading models, fluctuated dramatically during the three-month recording periods. Increases in sound pressure level appeared to be largely driven by large vessel activity, and accordingly exhibited a significant positive correlation with the number of Automatic Identification System tracked vessels at the two of the three sites. The near constant high levels of low frequency sound and consequential reduction in the communication space observed at these recording sites during times of high vocalization activity raises significant concerns that communication between conspecifics may be compromised during critical biological periods. This study takes the first steps in evaluating these animals’ communication spaces and alteration of these spaces due to anthropogenic underwater sound.

## Introduction

Sound is an efficient way to communicate in the marine environment, and animal inhabitants and people alike have developed ways to exploit this fact. Many organisms occupying the oceans actively use and produce sound. Marine mammals use sound as a primary method for communicating underwater over large distances, over shorter spatial scales fishes do the same. Marine invertebrates produce sound both actively for behavioural display purposes as well as passively due to feeding or movement. Features of ambient sound are a result of the characteristics of all of the contributing sound sources, including those composed of biological sounds such as animals vocalising (biotic), physical sounds, such as wind and water movement (geophysical or abiotic) and anthropogenic sounds such as shipping or construction (anthropogenic)^1^. Many marine organisms utilize ambient sound to navigate, choose their settlement or residence location, and to modify their daily behaviour, e.g., breeding, feeding and socializing^2–4^. Due to these reasons, ambient underwater sound is an important feature of marine habitats.

Anthropogenic sound in certain ocean regions has increased considerably in recent decades due to various human activities such as resource acquisition, global shipping, construction, sonar, and recreational boating^1^. As ocean sound increases, so does the concern for its effects on populations of acoustic signallers, making this a topic of significant scientific research focus. Effects of anthropogenic sound exposure can be seen in the physiology and behaviour of a range of marine organisms, from invertebrates^5,6^ to marine mammals^7^, with studies on these effects to date largely focusing on high-amplitude sources. Sound exposure can cause temporary hearing loss and threshold shifts^8^, reduction in temporal resolution ability^9^, damage and hair cell death in the inner ear^10,11^, and stress responses^12^. However, few studies have addressed the effects of lower-level and chronic sound exposures^13,14^.

Fishes represent over half of all vertebrate species, and more than 800 species from greater than 100 families are known to produce sound^15^. Not surprisingly these families have evolved a large diversity of sonic organs and sound producing mechanisms^16^. This variety of mechanisms has led to the production of diverse vocalizations and acoustic characteristics between species and populations. Fish vocalizations are an important component of the marine soundscape^17,18^ and they provide valuable information regarding the behaviour of the signaller in a variety of different contexts, such as general interactions, territorial displays, feeding, contact vocalization, and courtship interactions^19^.

Fishes exhibit an array of reproductive and social behaviours and the majority of species live fairly close to the coast or in fresh water environments, consequently they are exposed to the various human activities which produce sound^20^. In addition to the increasing amount of anthropogenic sound in the marine environment, these sounds often have prominent frequencies which fall within the frequency range of acoustic communication signals, therefore, having the potential to reduce communication efficiency. One of the most widespread, yet poorly understood means in which fishes could be affected by chronic, lower-level anthropogenic sound, such as vessel sound, is through the disruption of acoustic communication by masking^21,22^. In this situation, the receiver experiences an increase in the threshold of detection or discrimination of the signal which could potentially lead to complete or partial loss of received signal, misinterpretation of the signal, and/or subsequently changes in the response^21,23^. Although there is a growing body of literature on how signallers may avoid masking from anthropogenic sound, much of the research has been conducted on terrestrial organisms and marine mammals. To date, there have been very few documented studies on the potential of anthropogenic sound to mask, disrupt or reduce acoustic communication in fishes, and fewer still on the means of avoiding masking in the presence of extraneous sound^24,25^. Assessing the impacts of anthropogenic sound on the ecology of fishes is not the only concern. Fish provide livelihoods to hundreds of millions of people, and is a primary source of protein for >1 billion people worldwide with growth expected for more than 9 billion by 2050^26^, which is a difficult target without strict and ongoing management.

The central topographical feature within the Gerry E. Studds Stellwagen Bank National Marine Sanctuary (SBNMS), Stellwagen Bank itself, has supported high catch rates of Atlantic cod (*Gadus morhua*) and haddock (*Melanogrammus aeglefinus*) for centuries, and includes past knowledge of predicable spawning areas for cod within the sanctuary and greater Massachusetts Bay. Gulf of Maine cod stock contains genetically distinct spring- and winter-spawning subpopulations, and recent studies have highlighted waters both inshore and within the sanctuary as supporting seasonal spawning activity^27^. The spawning components of the Gulf of Maine cod stock are overfished, with the population at a historic low of about 82% less (winter stock) and 77% less (spring stock) than the same populations a decade ago^28^. The Gulf of Maine haddock stock is currently considered stable, and fishing quotas have recently been dramatically increased for this species due to an increase in stock size and to compensate for tighter controls on ground fish like cod^29^. The sanctuary and greater Massachusetts Bay waters also support the spawning activity of haddock, with major spawning locations on Stellwagen Bank occurring from January to May, usually peaking in February to April^30^. In addition to supporting these biologically important habitats, the sanctuary experiences high anthropogenic activity and subsequently increased levels of ambient underwater sound, particularly due to commercial shipping with a Traffic Separation Scheme running through its centre.

The purpose of this investigation was to examine the ambient soundscape (up to 1000 Hz) at three sites, two within and one inshore of SBNMS, which have been documented to support spawning activity for the Gulf of Maine cod and haddock stocks. These data were then used to calculate the estimated effective vocalization radius for each species in these areas during spawning time periods. These results take the first steps in assessing these animals’ communication spaces and the alteration of these spaces due to varying levels of background sound.

## Results

### Vocalization characteristics

**Figure 1:**
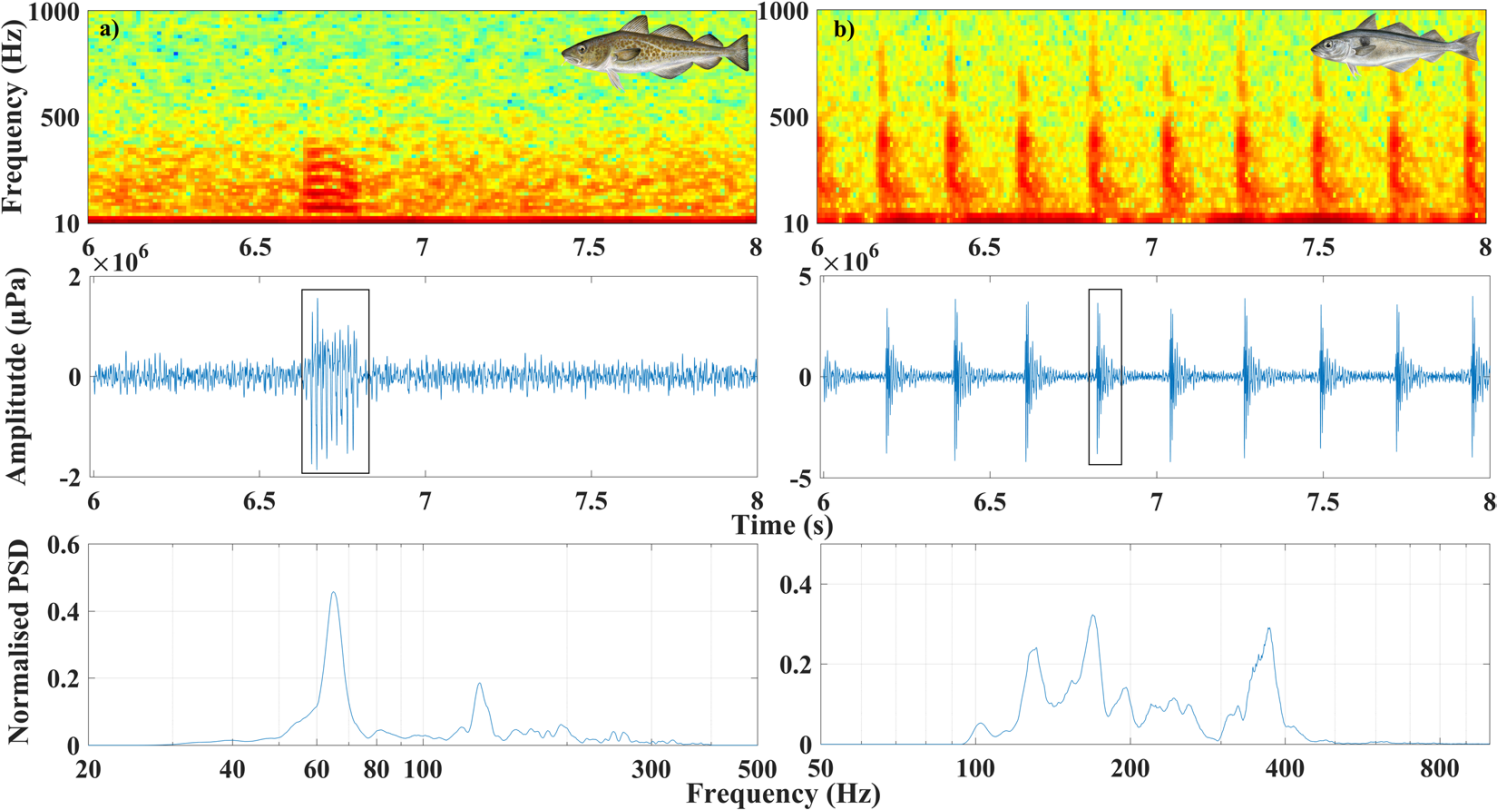
Panels showing acoustic characteristics of a spawning vocalization of a representative (**a**) an Atlantic cod grunt recorded within the three recording sites, and (**b**) Haddock knocks. Top panels: spectrogram of vocalizations, indicating frequency range. Middle panels: wave form of vocalization. Bottom panels: normalized power spectral density (PSD). Sounds were band pass filtered between 30 and 500 Hz for Atlantic cod and 100–1000 Hz for Haddock during the boxed pulses for the normalized PSD. Spectrograms and power spectra were computed using a 512-point fast Fourier transform (FFT), Hann-window, 80% overlap. Fish images with full permission by Scandinavian Fishing Year Book.

Vocalizations from Atlantic cod and haddock were present during the three-month recording period at each of the study sites. Atlantic cod grunts and haddock knocks during these recording periods were consistent with previously reported spawning vocalizations, with frequency and time-based measurements matching previous studies^31,32^. In the Atlantic cod spawning sites, ‘grunts’ were present for 100% (spring) and 83% (winter) of the days within the three-month sampling period. In the haddock spawning site ‘knocks’ and variations of the knock were present for 62% of the days within the three-month period (January 88.5%, February 75% and March 50%). Atlantic cod “grunts” (n = 40) had a mean peak fundamental frequency (f_1_) of 53 Hz (range = 41–69 Hz), mean duration of 232 ms (159–541 ms) and a mean number of pulses of 9.2 pulses per grunt (range = 7–11). Haddock “knocks” consisted of several arrangements including short slow knocks, short fast knock, long slow knocks, and long fast knocks (Fig. 1a). Haddock “knocks” (n = 40) had a mean peak frequency of 258 Hz (range = 184–356 Hz), mean sound duration of 6.3 s (range = 389 ms – 36 s), and mean number of knocks of 15.4 (range = 3–132) (Fig. 1b).

### Ambient sound levels

**Figure 2:**
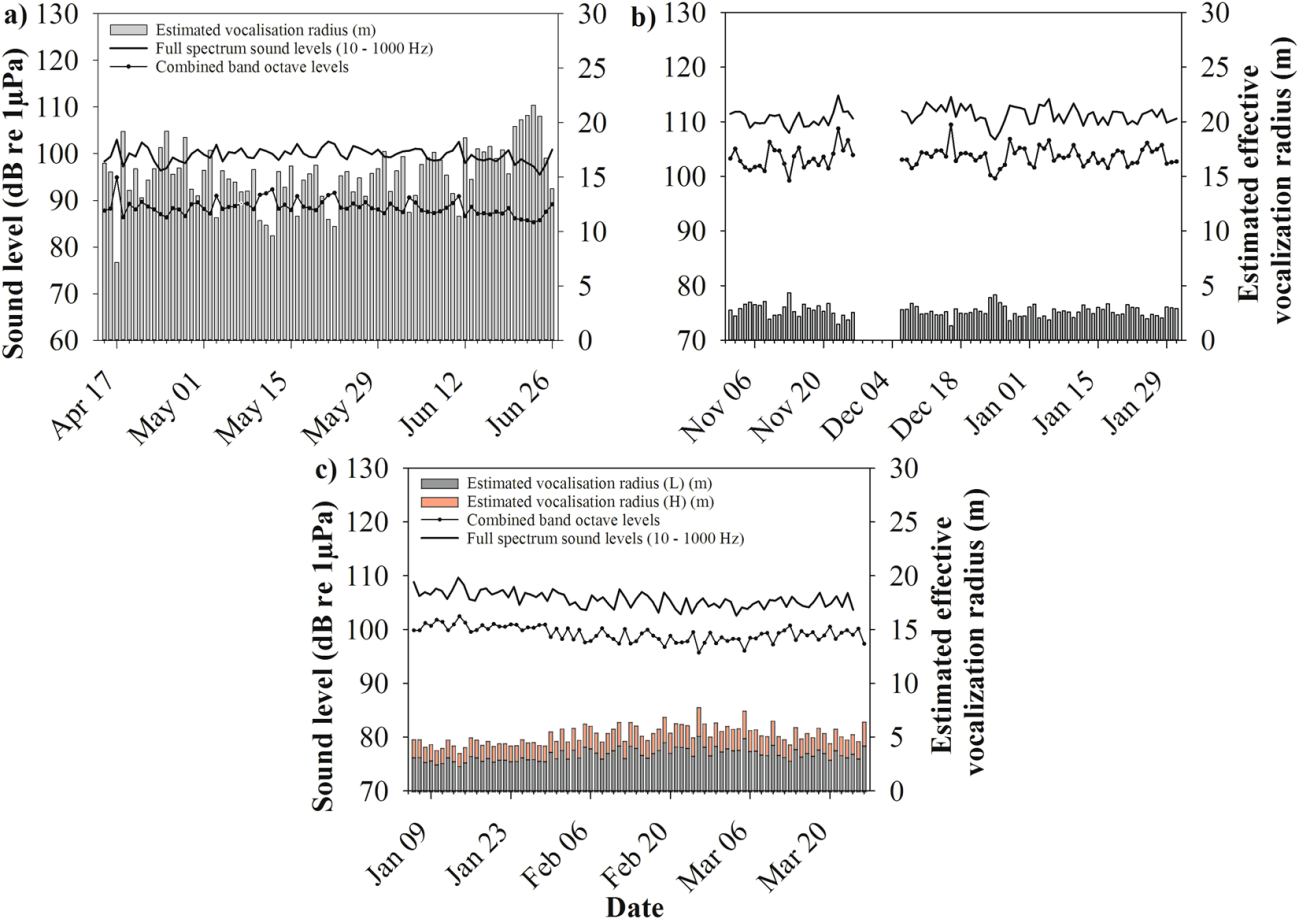
Multiplot showing daily means of full spectrum and combined band octave sound pressure levels (SPL_RMS_), and estimated effective vocalization radius (m). (**a**) Atlantic cod spring spawning site, (**b**) Atlantic cod winter spawning site, break in graph indicates a period of no acoustic data, (**c**) haddock winter spawning site. Combined band octave levels for both the winter and spring Atlantic cod spawning include bands 1 & 2, and for the haddock winter spawning site include bands 3 & 4 to best match vocalization frequency (see methods for f_bw_).

Ambient sound levels at the three study sites ranged from 84.7 to 139.9 dB re 1 µPa in the full spectrum band (10–1000 Hz) and 78.8 to 137.7 dB re 1 µPa in the combined octave bands (1,2 & 3,4) (Table 1, Fig. 2). Both full spectrum and combined octave band (matched with species vocalization) ambient sound pressure levels differed significantly among sites over the three-month sampling period (full spectrum: Kruskal-Wallis; *H* = 12128, *P* < 0.001, combined octave band: *H* = 13518, *P* < 0.001).

**Table 1:** Summary statistics of full spectrum (10–1000 Hz) and combined octave band sound pressure levels, AIS data, and estimated effective vocalization radius from each of the three recording sites over the three-month recording period.

Site		Spring Cod	Winter Cod	Winter Haddock
Full spectrum SPL dB re 1 µPa	**Min**	84.7	98.2	96.7
	**10** ^**th**^	94.1	105.1	100.7
	**Median**	99.3	109.8	104.8
	**Mean**	99.7	111.1	105.6
	**90** ^**th**^	105.5	117.3	110.7
	**Max**	125.3	139.9	132.8
Combined octave band levels dB re 1 µPa	**Min**	78.8	92.2	87.8
	**10** ^**th**^	82.6	97.7	94.9
	**Median**	88.7	102.3	99
	**Mean**	89	103.6	99.5
	**90** ^**th**^	95.1	111.2	104.2
	**Max**	120.6	137.7	127.6
Mean AIS vessel tracks/day in 10 nm radius		14.5	7	2.7
Effective vocalization radius (m)	**Min**	7.2	1.3	1.2 (L) 2.4 (H)
	**10** ^**th**^	11.3	2.1	1.5 (L) 2.9 (H)
	**Mean**	15.3	2.7	1.8 (L) 3.5 (H)
	**90** ^**th**^	19	3.4	2.2 (L) 4.2 (H)
	**Max**	21.6	4.4	2.7 (L) 5.2 (H)

The Atlantic cod winter spawning site had both the highest mean full spectrum sound level (111.1 dB re 1 µPa) and combined octave band levels (103.6 dB re 1 µPa) of all the recording sites over the three-month spawning period, as well as the highest 10^th^ and 90^th^ percentiles respectively (Full spectrum; 105.1 and 117.3 dB re 1 µPa, Combined bands; 97.7 and 111.2 dB re 1 µPa) and maximum sound level of 139.9 dB re 1 µPa which was due to a large vessel transiting over the site (Table 1). The Atlantic cod spring spawning site had both the lowest mean full spectrum sound level and combined octave band levels (99.7 and 89 dB re 1 µPa respectively) of the three recording sites, as well as the lowest 10^th^ and 90^th^ percentiles respectively (Full spectrum; 94.1 and 105.5 dB re 1 µPa, Combined bands; 82.6 and 95.1 dB re 1 µPa).

**Figure 3:**
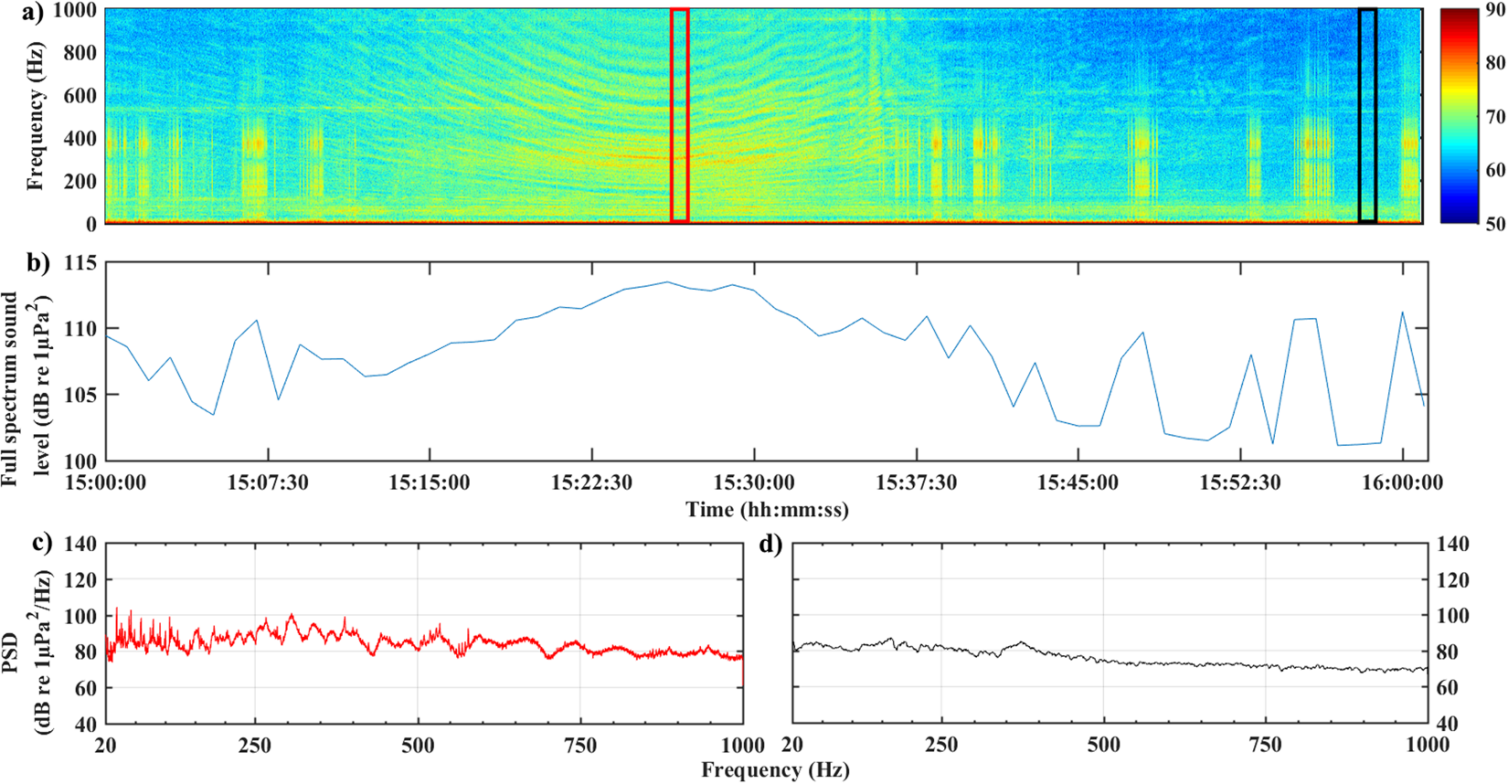
Example of visual representation of 1-hour vessel passage and haddock vocalizations at haddock winter spawning site. (**a**) spectrogram of 1-hour vessel passage, (**b**) Full spectrum sound level over 20–1000 Hz frequency range, (**c**) Power spectra of 20 sec length of recording when vessel is at its closest approach to hydrophone with a full spectrum sound level of 113.5 dB re 1 µPa in the 10–100 Hz frequency range (>90^th^ percentile), (**d**) Power spectra of 20 sec recording when vessel left immediate vicinity of hydrophone with a full spectrum sound level of 101.2 dB re 1 µPa in the 10–1000 Hz frequency range (50^th^ percentile). In figure a)  indicates time section for plot c and ◽ indicates time section for plot d. Colour bar units are dB re 1 µPa^2^ Hz^−1^. FFT: 1024, Hann window, 80% overlap.

The haddock winter spawning site had intermediate mean full spectrum sound levels (105.6 dB re 1 µPa) and combined octave band levels (99.5 dB re 1 µPa) over the three-month spawning period, as well as intermediate percentiles (Figs 2 & 3).

The pairwise multiple comparison demonstrated the Atlantic cod winter site had the highest full spectrum sound pressure levels (Dunn’s; vs. haddock winter *Q* = 54, *P* < 0.001, vs. Atlantic cod spring *Q* = 111, *P* < 0.001), followed by the haddock winter site (vs. Spring cod *Q* = 58, *P* < 0.001), and the Atlantic cod spring site had the lowest sound levels over the three-month period. The differences in combined octave band sound levels among sites followed the same pattern as the full spectrum levels, with the Atlantic cod winter site having the highest sound levels (Dunn’s; vs. haddock winter *Q* = 36, *P* < 0.001, vs. Atlantic cod spring *Q* = 114, *P* < 0.001), followed by the haddock winter site (vs. Atlantic cod spring *Q* = 79, *P* < 0.001) and finally the Atlantic cod spring site (Table 1, Fig. 3).

### Effective vocalization radius estimation

As the estimated effective vocalization radius was calculated by integrating the varying levels of ambient sound over the duration of the sampling period, these ambient levels influenced the effective vocalization radius greatly. The estimated effective vocalization radius at the three study sites ranged from 1.2 to 21.6 m (Table 1, Fig. 2), with significant differences among the three sites (Kruskal-Wallis; *H* = 273, *P* < 0.001).

The Atlantic cod spring site had the greatest mean effective vocalization radius of 15.3 m. The vocalization radius was less than 11.3 m 10% of the sampling period, or seven out of 74 days, and 19 m or less for 90% of the sampling period, or 67 of the 74 days. The winter Atlantic cod site had a much lower estimated effective vocalization radius than its spring counterpart with a mean of 2.7 m. The radius was 2.1 m or less for eight days, 10% of the sampling period, and was no greater than 3.4 m, 90% or 75 of the 84 sampling days.

For the haddock winter spawning site, two source levels (SL) were used to estimate the effective vocalization radius, a low SL and a high SL (for rationale see methods section). Of all four vocalization estimates, the lower estimate for haddock winter (L) had the smallest mean estimated vocalization range of 1.8 m. The estimated vocalization radius was 1.5 m or less for eight of the 82 sampling days (10%) and 2.2 m or less for 74 of the 82 days.

### Automatic Identification System Vessel Tracking and relationship with ambient sound levels

**Figure 4:**
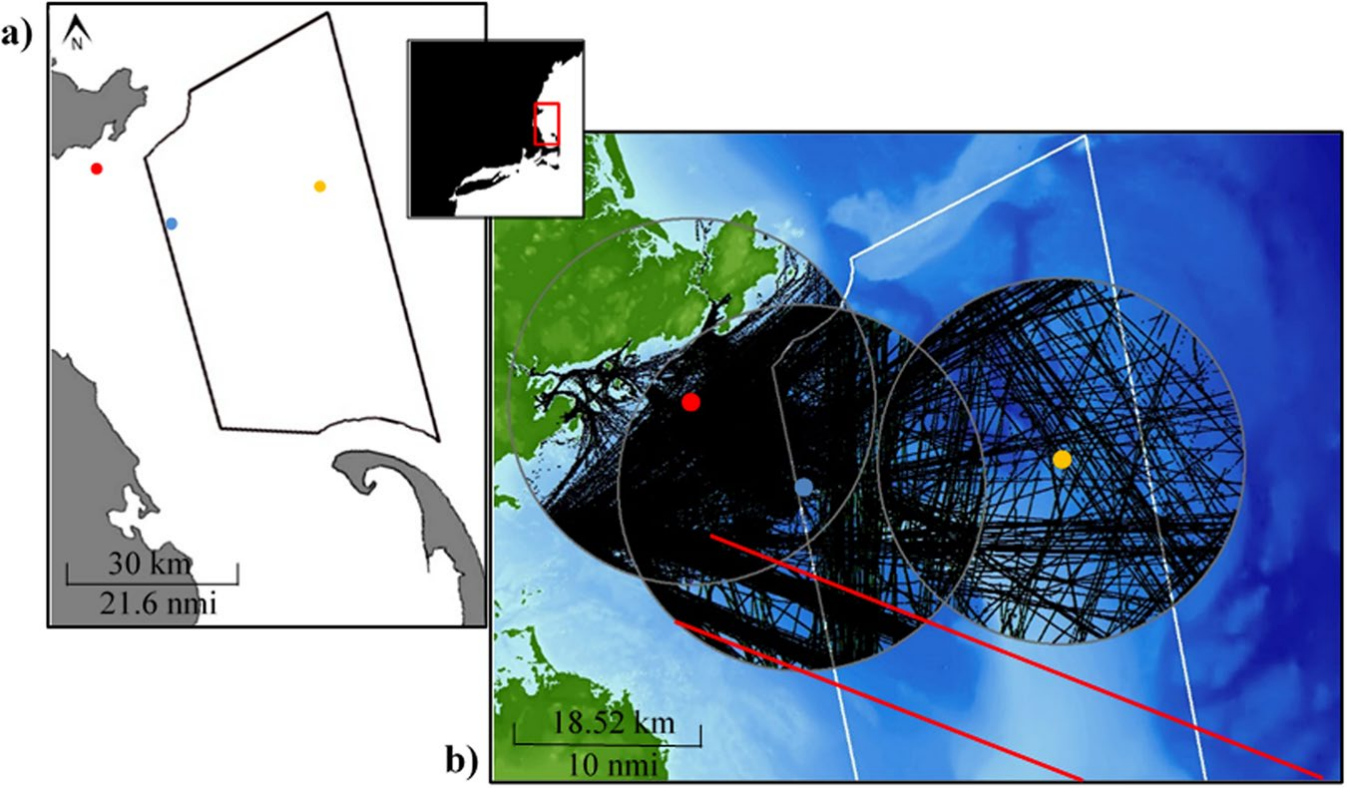
Maps showing (**a**) locations of recording sites within Massachusetts Bay and Stellwagen Bank National Marine Sanctuary in relation to the adjacent northeast coast of the United States, (**b**) AIS vessel tracks over the three-month recording period for both the Atlantic cod winter spawning site and the haddock winter spawning site within a 10 nm radius. Polygons marks the boundaries of Stellwagen Bank National Marine Sanctuary.  location of the Spring Cod Conservation Zone, the site of the Atlantic cod spring spawning recording location.  location of the Atlantic cod winter spawning recording location.  location of the haddock winter spawning recording location.  Boston traffic separation scheme. Maps created in ArcMAP 10.3.1 http://desktop.arcgis.com/en/arcmap/.

To further understand the contribution vessel sound had on the ambient soundscape at the recording site, the relationship between the daily numbers of AIS tracked vessels within the 10 nm radius and the daily combined octave band sound pressure levels were tested for a correlation (Fig. 4).

**Figure 5:**
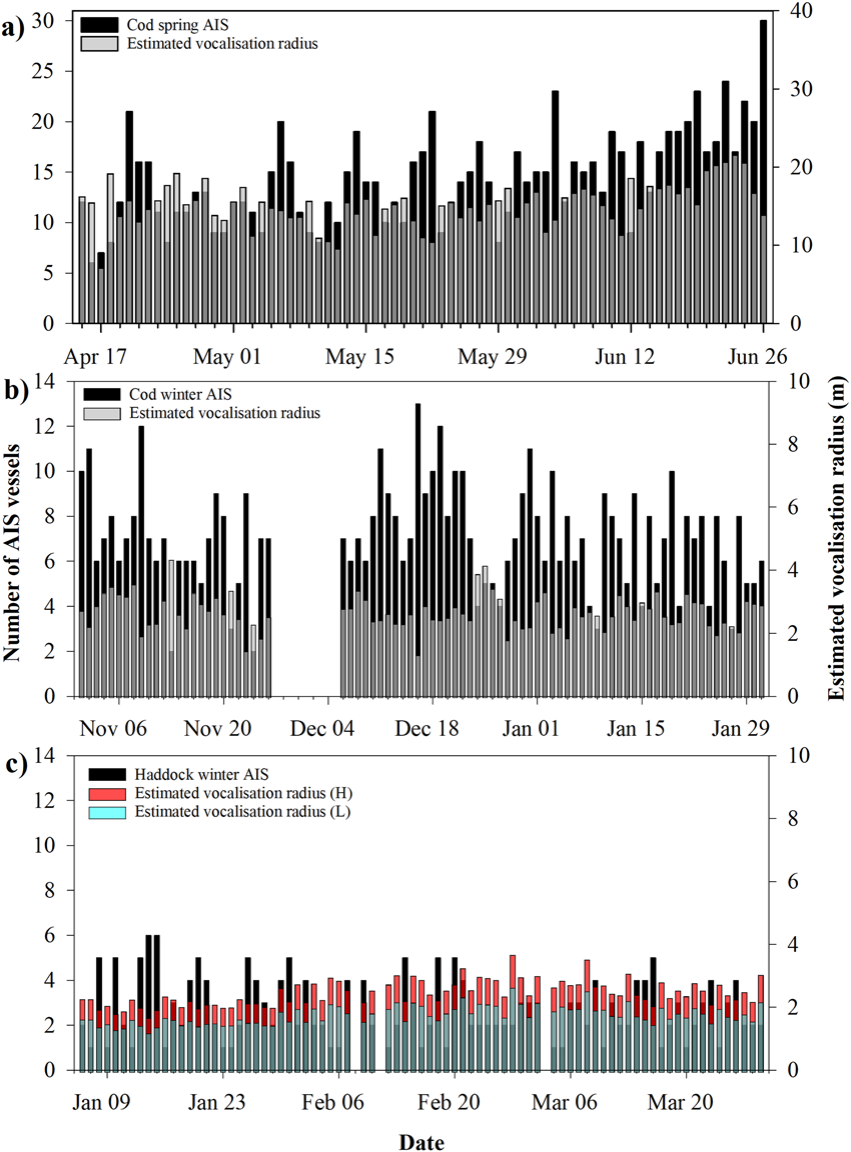
Bar graphs showing daily means of the estimated effective vocalization radius (m) and the daily number of AIS tracked vessels within a 10 nm radius of the recording site. (**a**) Atlantic cod spring spawning site, (**b**) Atlantic cod winter spawning site and (**c**) haddock winter spawning site, with H and L indicating effective vocalization radius calculated with high and low haddock source levels respectively. The breaks in graph indicates a period of no AIS data. Note different axis scales.

There was a significant difference in daily number of AIS tracked vessels within a 10 nm radius between the Atlantic cod winter, Atlantic cod spring and haddock winter spawning sites (Kruskal-Wallis; *H* = 184.9, *P* = < 0.001). The Atlantic cod spring spawning site had the greatest number of AIS tracked vessels with a mean of 14.5 vessels per day, compared to means of 7 and 2.7 per day at the Atlantic cod winter and haddock winter spawning sites (Table 1, Figs 4 & 5).

For the Atlantic cod winter spawning site, there was a statistically significant positive correlation between the daily number of AIS tracked vessels and the daily combined octave band sound levels; when the number of daily AIS vessels increased, the combined octave band sound levels increased (Pearson correlation; *r* (79) = 0.543, *p* < 0.0001). Since the effective vocalisation radius was calculated using the ambient sound levels, there was also a significant negative relationship between the number of AIS vessels and the daily estimated effective vocalization radius (*r* (79) = −0.544, *p* < 0.0001). A similar relationship occurred at the haddock winter spawning site: there was a statistically significant positive correlation between the daily number of AIS vessels and the daily combined octave band sound levels (*r* (78) = 0.509, *p* < 0.0001), and consequently a statistically significant negative relationship between the daily number of AIS vessels and the daily estimated effective vocalization radius (high source level: *r* (78) = −0.495, *p* < 0.0001, low source level: *r* (78) = −0.495, *p* < 0.0001). However, the Atlantic cod spring spawning site showed no significant relationship between the daily number of AIS vessels and the daily combined octave band sound levels (*r* (72) = −0.129, *p* = 0.279) or the daily estimated effective vocalization radius (*r* (72) = −0.124, *p* < 0.301).

## Discussion

Rising levels of anthropogenic underwater sound is of mounting concern in all marine environments. While high intensity sources hold much of research and management attention, more moderate sounds of much longer duration, like those produced by commercial shipping vessels, dominate background noise conditions over much larger areas and thus have the potential to effect greater numbers of marine animals. The results from the present study illustrated that ambient sound across the Atlantic cod and haddock spawning sites varied significantly, and as a result so too did the estimated effective vocalisation radius. These spaces were extremely reduced in the presence of sound produced by large vessels and at times the vocalisations of fin whales.

Both the “grunt” and “knock” vocalizations emitted by Atlantic cod and haddock occupy the same frequency range as many underwater anthropogenic sound sources^33^, with the peak of acoustic energy in the 50–260 Hz frequency band^34,35^. Field and laboratory measurements have shown that this bandwidth contains the range of the greatest acoustic sensitivity in both species^36,37^. The mean effective vocalization radii for spawning Gulf of Maine cod were estimated to be 2.7 m in winter and 15.3 m in spring spawning locations. Similarly, over the three-month winter spawning sample, Gulf of Maine haddock mean effective vocalization radii were between 1.8 m (low SL) to 3.5 m (high SL). The difference in effective vocalization radius between cod spawning locations appeared to be largely driven by the presence of large vessel activity in the surrounding environment, e.g., in the Atlantic cod winter spawning site the effective vocalization radius was as low as 1.3 m when there was a total of 13 AIS tracked vessels within a 10 nm radius of the recording site, and as high as 4.4 m in the presence of two AIS tracked vessels.

*There is no baseline information available on the distances cod and haddock have evolved to use acoustic signals*. It would be informative to examine masking under a range of different conditions in which they spawn, including other populations or sites with lower vessel traffic. Unfortunately, there are very few locations known where these populations spawn that are not heavily impacted by humans and heavily targeted as a fishery resource. Atlantic cod and haddock are known to exhibit complex “lekk” spawning behaviour, whereby males arrive to spawning grounds first and form dense aggregations over a small area and compete for dominance and females, via courtship displays, acoustic communication and aggression towards rivals. Females visit the aggregation, select a dominant male, initiate a spawning event and return to previous locations^38,39^. Therefore, females are not in continuous contact with males during the spawning season, and an attraction cue is essential for courtship^40^. Vertical and horizontal separation between males and females in spawning locations have been reported for several populations of Atlantic cod in the wild^40,41^. Haddock vocalization behaviour in the spawning season indicates that acoustic signals may also be used as a medium range signal to mediate the migration or attraction to spawning locations in transient populations, and not only over short distances (0–10 m)^42–44^. Male haddock have often been observed to repeat long knocking vocalizations for hours at a time, often in solitary display with no other fish close by^35,45^. This behaviour indicates that the male is occupying a home range or territory where it is exhibiting unambiguous sexual readiness to females^35^. It is also hypothesized that the chorus of large aggregations of male Atlantic cod at spawning locations may serve as a long-range signal attracting females to the area^46^. If the signal or chorus is undetected or misinterpreted due to masking it could lead to the mistiming or unsuccessful location of spawning aggregations, which is critical to the survival of these managed populations.

*Mounting evidence suggests that acoustic communication can affect the survival and reproductive success of fishes, including direct evidence for Atlantic cod* ^47^. The Gadidae family contain several vocal species, where the sounds produced are species specific and usually relatively simple. However, haddock, produces a variety of knock sounds which are used in a diverse range of behavioural contexts^31,38^. Evidence suggests that haddock vocalizations serve to not only get male and female fish together in a specific part of the ocean but also play a key role in synchronizing the reproductive behaviour in males and females^35^. Unlike haddock who have a wide acoustic repertoire, Atlantic cod are thought to be less versatile vocalists during courtship, they produce single “grunts” which are believed to function as both an agonistic display but also to be especially significant as a reproductive advertisement and used during spawning^48^. If anthropogenic sound reduces the efficiency of the vocalizations utilized by these species, this interference could potentially impact their reproductive success and survival through the incorrect assessment of the quality of potential mates or competitors, reduction in the ability to attract mates and/or the mistiming of gamete release.

Although the behavioural effects of masking are often difficult to measure, other quantifiable effects of anthropogenic sound on the reproductive and developmental physiology of Atlantic cod have been documented^14,49^. Sierra-Flores *et al*., (2015) demonstrated that a daily randomized 60-minute exposure to a linear sweep (100–1000 Hz @ 132 dB re 1 μPa) over a two-week period resulted in a significant reduction in total egg production and fertilization rates, reducing the total number of viable embryos by over 50% compared to a control. Effects of anthropogenic sound are also not limited to the adult population, Nedelec *et al*. (2015) revealed that for newly hatched Atlantic cod two days of exposure to both regular and random noise from ships reduced growth, and led to faster yolk sac utilization. After 16 days, fish exposed to regular ship noise had reduced body width-length ratios and were easier to catch in predator avoidance experiments^14^.

*Several studies conducted in the field on marine teleosts (including cod and haddock) have confirmed that masking of a signal can occur under relatively quiet background sound conditions* ^36,50^. These studies demonstrated that hearing thresholds increased with decreasing frequency separation between the signal and the masking sound band^51^. As with many other organisms, fishes evolved in environments with varying levels of background ambient sound, they have regularly encountered loud sources of naturally occurring biotic and geophysical sounds including wind, rain, the action of waves at the surface of the water. There are also several examples that illustrate both hetero- and conspecific sounds have the potential to overlap in both the frequency and time domain and therefore have the potential to mask communication^52–54^. Furthermore, several solutions, “masking releases”, to ensure the audibility of a signal over the background noise have been observed in other taxa and have been suggested could be occurring in fishes. The simplest way to avoid the impacts of a potential threat is to avoid it; however, when applying this to underwater sound it is certainly not always possible. This is especially true if a species is dependent on a certain area for critical resources, or with sources whose sounds dominated certain biologically relevant frequencies and have long-distance propagation properties, such as the low frequency sound produced by large vessels^55^. These limitations apply to both populations in this study and particularly for spawning components of Gulf of Maine cod in Massachusetts Bay, as they are known to exhibit extremely site fidelity returning to the same spawning locations year after year^56^. There is also evidence for directional masking release which could allow for the detection and discrimination of a signal in the presence of another. Although the particle velocity component of the sound field was not measured in the current study, it may prove to be important when addressing short-range communication and masking in these species. In some cases, the sound pressure and particle velocity components of the sound field are directly related, however, in relatively shallow waters this is not the case, and deviations between these two components can be especially high in the near field (close to the sound source). Although one’s ability to detect vocalisations wasn’t addressed in data of the current study, this component may be a factor resulting in vocalisations being detectable at different ranges than those calculated by pressure based equations^57^. Additionally, the particle velocity component encountered in the nearfield may give listeners information on the directionality of the sound sources when the signal of interest and the competing sound are received from differing directions. This may allow the listener to utilize binaural timing differences to enhance discrimination^58^. This has been briefly investigated in a few marine mammal species^59,60^, and could be possible in fishes, however, the extent of the masking release is unknown^37^.

Temporal avoidance is another potential solution used by signallers in the presence of high background sound. Here a signaller could take advantage of intrinsic gaps or fluctuations in the competing signals. However, this strategy is not always possible in species using acoustic communication during critical time periods due to daylight, tidal or moon phase synchrony, and especially in the case of locations where the low frequency sound from large commercial vessels is the competing signal. In this case there is often no predicable time of the day where vessel noise is not present, or in some locations where distant low-frequency vessel sound is a constant. Temporal adjustments to the communication signal itself, such as increasing the duration of a brief signal has also shown to considerably enhance its detectably^61^. In the case of longer acoustic signals, an increase in duration or redundancy by repetition can subsequently increase the probability that part of the signal will be received during a period of quieter background sound^62,63^. This mechanism seems plausible for some fish species, especially those who exhibit complex or repetitive vocalization structure, such as haddock. Conversely, this may not be possible for species with simple or singular vocalization structure, such as Atlantic cod. With high interspecific diversity, temporal patterns of sounds are thought to be the most crucial factors in carrying information during acoustic communication in many teleost fishes, with receivers extracting information from pulse number, duration and repetition rate^64,65^. Consequently, population wide adaptations in temporal signal patterns could certainly occur across evolutionary time, but seem unlikely to occur over the current time scale of increasing background sound. There has been less than a handful of studies that have shown fish species capable of altering their vocalization signals in the presence of anthropogenic sound, and those that have, *Cyprinella vesta* and *Opsanus tau*, have been found to alter the power of their call (Lombard effect) while also changing behavioural traits such as distance between signalers^66,67^.

*In the current study, ambient sound levels fluctuated greatly within recording sites, occurring on both an hourly and daily time scale (85–143dB and 79–138dB re 1* *µPa full spectrum and combined octave bands respectively)*. In support with previous studies, large vessel activity around the recording sites appeared to be a predominant contributor to increases in the ambient sound levels, especially in the acoustic bands occupied by the vocalizations, and regularly increased the ambient background sound by at least 10 dB (re 1 µPa in the 20–1000 Hz range)^68,69^. The Atlantic cod winter spawning site had the second highest AIS-monitored traffic, it also had over double the acoustic power (≥3 dB) more than 50% of the time in both full spectrum and combined band octave sound levels compared to the least trafficked recording site. Accordingly, there was a significant positive correlation between the combined octave band sound levels and the number of AIS vessels present within a 10 nm radius at both Atlantic cod and haddock winter spawning sites. There were also increases in the ambient sound levels, and consequently a temporary reduction in the correlation, due to the vocalizations of baleen whales, particularly fin whales who produces both 20 and 40 Hz pulses^70^, and to a lesser degree fishes, however, their influence on local acoustic conditions at the sites was less pervasive and their presence did not affect the correlation. Conversely, there was no significant correlation between the two factors at the Atlantic cod spring spawning site. There are several possible reasons for this; the number of AIS tracked vessels was larger at this site, this was predominately because the data was collected five years subsequent to the other sites. In that time, it is estimated there was at least a 30% increase in the number of vessels with AIS installed in the region (US Coast Guard, 2017). The number of smaller sized components of traffic installed with AIS transmitters was increasing, consequently a higher proportion of the total vessels present in the recording area were registered, but with significant differences in the amount and type of sound they emitted relative to the large ocean-going commercial traffic highly represented at the other two sites^33,71^. When examining the size class of AIS tracked vessels at this site there were higher numbers of vessels <40 m in length compared to the other two sites. The total number of AIS vessels quantified at the spring spawning location had subsequently less impact on the combined octave band sound levels at this site. The site’s coastal proximity, proximity to many recreational marinas and to the Boston vessel separation scheme, and sampling season relative to the other sites also likely differentiate its vessel activity, and the sound signature of that activity, from the two more offshore locations. For these reasons, AIS data is not a perfect representation of sound that could potential mask other biological signals in all environments, and care must be taken when using AIS data to infer masking potential.

*The results of the current study have taken the first steps in assessing the direct influence of anthropogenic sound on the communication spaces of two ecologically and commercial important fish species at three locations highly influenced by human activities*. It highlights the ever-increasing need to better understand the role anthropogenic sound has in the disruption of intraspecific acoustic communication. Future research should focus on examining the extent to which specific species can compete with anthropogenic noise through adaptation or adjustment of their acoustic signals, address the abilities to receive and interpret signals in the presence of another, increase the accuracy of vocalization propagation and detection models by incorporating oceanographic, and bathymetric variables, as well as updating species-specific source level measurements in the field. Further consideration on the use of multisensory cues and how they supplement each other is also needed to support our understanding of the behavioural and physiological effects of prolonged exposure to low frequency sound. This research also highlights the need to gain a better understanding of the spatial and temporal use of unique habitats that are predictably used for critical life history events in declining populations. Identifying and better understanding these consequences at lower trophic levels is important to advancing the management of shared acoustic space.

## Methods

### Instrumentation

The acoustic recordings were made using Marine Autonomous Recording Units (MARUs; Cornell University Bioacoustics Research Program^72^). At all recording sites, the units continuously sampled at a rate of 2000 Hz with a flat frequency response sensitivity (±1.0 dB) of ~151.2 dB re 1 μPa between 10 and 2000 Hz (HTI-94-SSQ, High Tech Inc., Gulfport, MS, USA).

### Deployments

Between January 2006 – February 2007 and April – June 2011, several marine autonomous recording units (MARUs; Cornell University Bioacoustics Research Program (Clark *et al*., 2002)) were deployed within the Sanctuary to calculate the spatial and temporal variability of soundscapes and to detect vocally active marine species. From these deployments, sites for the current study were determined using prior knowledge of spawning areas and spawning dates for the two Gadoid species; Atlantic cod (*Gadus morhua*) and haddock (*Melanogrammus aeglefinus*). Recordings were also examined for times of high vocal activity attributable to spawning behaviour (Stanley, Van Parijs & Hatch; in prep).

Three-months of recordings each from three different sites were chosen to represent spring and winter spawning periods for Atlantic cod and a winter spawning period for haddock. The Atlantic cod spring spawning site, 42°31′5.58″N, 70°41′43.26″W, occurred within the Spring Cod Conservation Zone (SCCZ), in 51.4 m of water in northern Massachusetts Bay, 5 km south of Gloucester, USA (Fig. 4). The SCCZ is a seasonal fisheries closure area established to attempt to provide protection for a historic and once predictable coastal cod spawning aggregation (http://www.mass.gov/eea/agencies/dfg/dmf/programs-and-projects/cod-conservation-zone.html). The substrate at this site was predominately fine-grained sediment, occasionally broken up by cobble and boulder deposits and larger bedrock structures^73^. Recordings were utilized from 15 April – 27 June 2011 during the spring spawning season^74^. The Atlantic cod winter spawning site, 42°26′29.69″N, 70°33′29.59″W, occurred within the SBNMS, in 49.3 m of water with a gravel dominated substrate. Recordings from 01 November 2006 – 31 January 2007 were utilized to represent the winter spawning season (Fig. 4)^75^. The haddock winter spawning site, 42°28′11.30″N, 70°14′32.82″W, also occurred within the SBNMS at a depth of 66.4 m. The substrate at this recording site was largely dominated by gravel, but also had areas of sand and cobble and/or boulder areas. Recordings from the 6^th^ January – 28^th^ March 2006 were used to best represent the winter spawning period^76^.

### Acoustic analysis

#### Vocalization characterization

Using previously used sound classification parameters^31,32,34,35^, the acoustic data from the three study sites were hand browsed for haddock in Raven Pro 1.5 (The Cornell Lab of Ornithology, NY, USA) and run through an Atlantic cod detection algorithm^77^ to ensure vocalizations from the two species were present during the selected recording periods. Forty randomly selected vocalizations from each species were selected during their spawning period, and summary statistics were taken including peak fundamental frequency, sound duration and number of pulses (cod), and peak frequency, sound duration, and number of knocks (haddock). Each day was examined and daily percentage presence of vocalizations for the specific species was calculated for the 3-month period at each site.

#### Ambient noise analysis

The ambient sound was measured over the entire three-month spawning period at each recording site. Using purpose-written MATLAB scripts, sound pressure levels for the full spectrum (10–1000 Hz with a 2 kHz sample rate) were calculated at 1 s resolution at each of the three sites, and daily metrics were also calculated for comparison (SPL; RMS, median, 10 & 90^th^ percentiles).

The precise bandwidths for the auditory filters of the species of interest are unknown, but have been described as being slightly larger than other vertebrates^78^. Thus, filters were approximated using octave filter banks. This method is considered more suitable to gauge the audibility of a signal in the presence of ambient noise. Using MATLAB scripts modified from octbank.m by Christophe Couvreur, octave band analyses were conducted at 1 s resolution to characterize the bands with centre frequencies (ƒ_c_) at 31.5 (ƒ_bw_ 22 – 44 Hz) (band 1), 63 (ƒ_bw_ 44 – 88 Hz) (band 2), 125 (ƒ_bw_ 88 – 177 Hz) (band 3), and 250 Hz (ƒ_bw_ 177 – 355 Hz) (band 4), additionally daily metrics were calculated for comparison (SPL; RMS, median, 10 & 90^th^ percentiles) over the all three-month periods at each site. Bands 1 & 2 were selected for Atlantic cod recording sites and bands 3 & 4 for the haddock recording sites as these bands best matched the frequency distribution of the vocalization types for each species. Daily sound pressure levels were calculated in the combined bands 1 & 2 (band 1,2) and 3 & 4 (band 3,4) for Atlantic cod and haddock respectively, for use in the effective vocalization radius calculation (ANL). All acoustic analysis was carried out in MATLAB R2015b (Mathworks Inc., USA).

#### Estimated effective vocalization radius

Using the modified sonar equation from Clark *et al*. (2009), adapted for the use with fishes^78,79^, the estimated effective vocalization radius was calculated for each day during the recording periods. This gave an estimated radius in which a single Atlantic cod and/or haddock vocalization could theoretically propagate under the ambient noise levels encountered over the three-month recording period.

For the purpose of this study we assumed; (1) signal detection was limited by ambient noise, (2) vocalization source level did not vary in response to varying ambient noise levels i.e., Lombard effect, (3) fish hearing had equal omnidirectional sensitivity, and (4) the sound source propagates approximately omnidirectionally.

1 $${\rm{SE}}={\rm{SL}}\,-\,{\rm{TLsp}}\,-\,{\rm{MSL}}\,-\,{\rm{DT}}$$

SE is signal excess, when SE = 0 it is routinely defined in respect to sonar systems as the 50% probability of signal detection^80^; SL is the source level of the fish vocalization at 1 m from the source – 127 dB re 1 µPa @ 1 m for Atlantic cod^46^, and as there is no published research on the source level of haddock vocalizations two levels were used from unpublished research findings, 119.2 and 125 dB re 1 µPa @ 1 m for haddock low and haddock high respectively (Hawkins; pers. comm.); MSL is the mean sound level for the site, calculated as the mean daily combined octave band level of sound (SL_RMS_) (band 12 or 34 for cod or haddock respectively) for each day in the three-month recording period for each site; TL_sp_ is the simplified spherical spreading transmission loss, calculated as 20 log[r (m)]^81^, the spherical spreading transmission loss model was used due the relatively low source levels of the vocalizations and the water depth at the site. The vocalisations are estimated to propagate over a shorter distance than the depth range of the water, therefore are assumed to propagate in a spherical manner. DT is the detection threshold, defined as the difference between the signal and the sound at a threshold where the signal can still be perceived by the recipient. There are no precise data for the detection threshold in fishes, therefore the current study used a detection threshold of 15 dB, which is considered conservative and attempts to incorporate the understanding of the masked detection thresholds of Atlantic cod and haddock^37,51,79^.

Effective vocalization radius (*r*, eq. 3) was derived from eq. 2 when SE = 0.

2 $${\rm{TLsp}}=20\,{\rm{log}}\,{\rm{r}}\,$$

TL_sp_ will give *r* when SE = 0, and therefore:

3 $$r=10({\rm{SL}}-{\rm{MSL}}-{\rm{DT}}/20)$$

### Automatic Identification System and Vessel Tracking

The sanctuary partnered with the US Coast Guard to gather early data from implementation of the Automatic Identification System (AIS) in Massachusetts Bay, providing high-resolution information since 2004 on the distribution and density of large commercial traffic through the Traffic Separation Scheme that transits the sanctuary accessing the Port of Boston.

The relationships between the daily number of AIS vessels within a 10 nm radius of the Atlantic cod and Haddock spring and winter spawning study sites, the daily sound pressure levels and the daily estimated effective vocalization radii were investigated to further understand and identify the acoustics drivers at each site. Following the methods of Hatch *et al*.^82^, AIS data collected during the study period were extracted and reformatted using AIS Miner (U. S. Coast Guard Research and Development Centre, 2005) and custom software written in Python V2.5.1. (Python Software Foundation, 2007) added to the NOAA data package^83^. The daily number of AIS tracked vessels within a 10 nm radius around the recording sites over the 3-month sampling period was calculated, excluding vessels with a ground speed of zero. This spatial extent was chosen following the rationale of Hatch *et al*. 2008, with an extended vessel radius. This radius would roughly estimate the area within which a ship with a source level of ≥180 dB re 1 µPa would ensonify the recording site at levels >116 dB re 1 µPa, therefore including sources positioned at a greater distance from the recording site while still rising above ambient sound levels at the sites (Table 1). The theoretical source level of 180 dB re 1 µPa was used because a large proportion of commercial shipping vessels are in the range of 170–190 dB^69,84^.

### Statistical analysis

For statistical tests, including detecting significant differences in ambient sound levels in the full spectrum and combined octave bands, estimating effective vocalization radii, and estimating the number of AIS vessels during respective spawning periods among sites, non-parametric statistical methods were used to test for differences among sites as the data had unequal variance among treatments and some data had a non-normal distribution^85^. To compare differences among sites, the Kruskal-Wallis test was used. If such tests provided significant results, a Dunn’s pairwise multiple comparison was used to isolate differences among individual sites. A Pearson Correlation test was performed to assess the relationship between the number of AIS vessels present per day and the combined octave band levels. All analyses were performed using SigmaPlot 13 (Systat Software Inc) and SPSS Statistics (IBM) Software.

### Data availability

The datasets generated during and analysed during the current study are readily available from the corresponding author on request.
